# Effect of a Novel Lavender Extract on Plasma Lipid and Lipoprotein Metabolism, Glucose Tolerance and Adipose Tissue Metabolic Activation: A Preclinical Safety and Efficacy Study

**DOI:** 10.3390/nu17010076

**Published:** 2024-12-28

**Authors:** Georgia Kakafoni, Evangelia Zvintzou, Smaro Kyroglou, Katerina Giannatou, Victoria Mparnia, Patroklos Vareltzis, Kyriakos E. Kypreos

**Affiliations:** 1 Pharmacology Laboratory, Department of Medicine, University of Patras, 26500 Rio Achaia, Greece; kakafonigeo@gmail.com (G.K.); liliazv@upatras.gr (E.Z.); katerinagiannatou001@gmail.com (K.G.); victoriamparnia2@gmail.com (V.M.); 2 Laboratory of Food and Agricultural Industries Technologies, Chemical Engineering Department, Aristotle University of Thessaloniki, 54124 Thessaloniki, Greece; kyrosmar@cheng.auth.gr (S.K.); pkvareltzis@cheng.auth.gr (P.V.); 3 Department of Life Sciences, School of Sciences, European University Cyprus, 2404 Nicosia, Cyprus

**Keywords:** lavender, lavender solid waste, *Lavandula stoechas*, efficacy, safety, lipoproteins, glucose homeostasis, white adipose tissue, brown adipose tissue, mitochondrial metabolic activation, circular economy

## Abstract

Background/Objectives: Lavender has been utilized for its medicinal properties since ancient times, with numerous health benefits reported. This study aimed to valorize solid waste from lavender essential oil production by developing a novel lavender extract from solid lavender residues. The extract’s preclinical safety and efficacy were evaluated with emphasis on plasma lipid and lipoprotein metabolism, glucose tolerance, and adipose tissue metabolic activity. Methods: Male C57BL/6 mice were divided into four groups of five mice each and fed for 30 days with lavender extract encapsulated in 10% maltodextrin, mixed with a standard chow diet. The first group (Lav 1×) received 21.1 mg/kg/day, the second group (Lav 10×) received 211 mg/kg/day, and the third group (Lav 100×) received 2110 mg/kg/day. A placebo group consumed the standard diet without lavender extract. Key outcomes included plasma lipid and lipoprotein profiles, transaminase levels, HDL antioxidant and anti-inflammatory potential, glucose tolerance, and mitochondrial activity in white (WAT) and brown (BAT) adipose tissues. Results: The novel lavender extract induced dose-dependent improvements in lipid and lipoprotein metabolism, glucose tolerance, and adipose tissue activity. The 2110 mg/kg dose (100×) demonstrated the most significant beneficial effects, although it was associated with a slight elevation in hepatic transaminase levels, indicating potential mild hepatic stress. **Conclusions:** Overall, the novel lavender extract exhibits promising health benefits with no major safety concerns at the tested doses, supporting its potential for therapeutic applications.

## 1. Introduction

Lavender (*Lavandula* sp.) belongs to the family Labiatae (Lamiaceae), and the genus *Lavandula* is native to areas bordering the Mediterranean Sea, the Middle East and Southwest Asia. In general, there are more than 30 species, dozens of subspecies, and hundreds of hybrids [1]. The wide distribution of lavender reflects its historical and cultural importance, as well as its widespread use, either as a medicinal plant or as an aromatic plant, in the daily life and traditions of many regions.

Lavender has many benefits for human health [2]. Its use for medicinal purposes has been known since ancient times and has been extensively studied. Some of the most typical traditional medicinal properties are: antioxidant, antifungal and antibacterial [3,4,5], cytotoxic [6] antiseptic, anti-inflammatory, and analgesic [3], anticonvulsant, anticholinergic, relaxing [2], and antidepressant [7] properties. In the past, lavender was used therapeutically for gastric ulcers, asthma, and Parkinson’s disease [2]. It has beneficial effects in the treatment of headache and symptoms associated with migraine episodes, while also reducing the spread of pain [8]. It has also been found to have beneficial effects on dementia as an adjunct therapy [9], improving memory in women with multiple sclerosis [10]. In addition, lavender essential oil improves the quality of sleep [11], alleviates symptoms of dysmenorrhea [12] and menopause [13], and helps enhance wound-healing processes [14]. Moreover, studies have shown that lavender has beneficial effects in anxiety disorder [15,16,17].

Lavender has also been proven effective in cardiometabolic diseases. Studies have shown that inhaling lavender essential oil can lower systolic and diastolic blood pressure and heart rate [18]. Also, data from rabbit studies suggest that it may reduce aortic cholesterol content and atherosclerotic plaque accumulation, thus providing a vasoprotective effect [19,20]. A study of 19 healthy medical personnel examined the effect of inhalation of lavender essential oil on endothelial function after night shifts. Flow-mediated dilation (FMD) of the brachial artery was reduced after the night shift but was significantly improved in subjects who had undergone aromatherapy for 30 min with lavender inhalation [21]. In another study, 30 healthy men who inhaled lavender essential oil showed a marked increase in Coronary Flow Velocity Reserve (CFVR) and decreased serum cortisol levels, a change not seen in the control group, demonstrating its potential efficacy in coronary traffic [20]. Furthermore, in another clinical study, lavender significantly reduced blood pressure compared to the control group during sleep [22].

Infusion and suspension of *Lavandula stoechas* L. (*L. stoechas*), induces hypoglycemia in normal rats, reaching maximal activity 30 min after administration [23]. Further studies with *L. dentata* and *L. latifolia* have shown that the active hypoglycemic components are partially water soluble. These extracts were ineffective in rats with alloxan-induced type I diabetes, indicating that intact pancreatic cells are essential for their pharmacological activity. The active ingredients have not been chemically identified [24]. In another study, the essential oil of *L. stoechas* showed a beneficial effect on glucose levels in rats with alloxan-induced diabetes. In addition, protective effects on diabetes-related kidney and liver damage and mitigation of adverse effects on lipid metabolism were observed [25]. Clinical data from 37 adult patients with type 2 diabetes showed no significant differences in fasting glucose after 4 weeks of aromatherapy with *Lavandula angustifolia* essential oil inhalation, 5 min before bedtime, compared to the control group [26].

The extraction process of this valuable natural product has been significantly improved during the last decades. The optimization of lavender oil extraction using both green energy sources (microwaves, ultrasound, supercritical CO_2_ or their combination) and alternative solvents (e.g., ionic liquids) instead of classical hydrodistillation and steam distillation is of great interest [27,28,29,30]. Recently, the complete valorization of aromatic herbs including hydrolats, leachates, and solid waste has become a major challenge in the pursuit of a circular economy and environmental impact reduction [31,32,33]. Vareltzis et al. (2024) [34] proposed an integrated process consisting of a green extraction method and a consequent drying process for the valorization of lavender solid waste. Forty-three percent of the total phenolic content was found in the lavender solid waste, while less than 1% was present in the essential oil fraction. Ultrasound-assisted extraction led to a higher phenolic content and greater antioxidant properties compared to microwave assisted extraction. Furthermore, the encapsulation of the extract using maltodextrin improved the drying process yield [34]. Other benefits of encapsulation include stability against oxidation, improved bioavailability, and masking of the characteristic odor of lavender essential oil.

The beneficial properties of lavender make it a candidate as a functional food ingredient. As part of our ongoing efforts to valorize aromatic herbs in the pursuit of a sustainable circular economy and environmental benefits, the aims of the present preclinical study were to evaluate the efficacy and potential toxicity of a novel maltodextrin encapsulated lavender extract isolated from the leaves of the plant after essential oil was removed. To evaluate the potential benefits of this extract as an active food ingredient for metabolic health, our studies focused on lipid and glucose homeostasis.

## 2. Materials and Methods

### 2.1. Raw Material

Τhe aerial parts of *Lavandula stoechas* were kindly provided by a local producer in Rodolivos (Rodolivos IKE, Serres, Greece).

### 2.2. Production of Dried Extract

Extraction and drying processes have been optimized and described in previous work [34]. Briefly, lavender leaves were submitted to hydrodistillation and the solid waste was dried at a temperature of 40 °C and ground using a laboratory mill (PX-MFC 90 D, KINEMATICA, Malters, Switzerland) and a 1 mm sieve. Afterwards, ultrasound-assisted extraction was conducted using an ultrasonic homogenizer (Sonopuls HD 4100, Bandelin, Berlin, Germany). One (1) g of dried lavender solid waste was mixed with 18 mL of ethanol–water solvent (1:1) and the ultrasound amplitude level applied was 40% for 10 min. After extraction, evaporation was performed in a rotary vacuum evaporator (Model R 114, Buchi Laboratoriums-Technik, Flawil, Switzerland) to remove ethanol, and 10% *w*/*v* maltodextrin was added. The solution was spray-dried using an ADL 311S spray-dryer (Yamato Scientific Co., Tokyo, Japan) with a maximum compressed air pressure of 0.1 MPa, a feed flow rate of 3.9 mL/min, a drying air flow rate of 0.07 m^3^/min, and an inlet air temperature of 170 °C. The resulting outlet temperature was about 57 ± 2 °C. The dried extract was collected and stored in heat-sealed plastic bags in a vacuum (MULTIVAC, C200, Wolfertschwenden, Germany) at −20 °C.

### 2.3. Characterization of Dried Extract

The moisture, bulk density, hygroscopicity, water activity, total phenolic content and antioxidant activity of the dried extract were the main properties determined following the methods described in a previous study [34]. The characteristics of the dried extract are presented in the Supplementary Materials (Tables S1 and S2).

### 2.4. Animals

Male C57BL/6 mice, approximately 24 weeks old, were allowed unrestricted access to water under a 12-h light/dark cycle (7:00 a.m.–6:59 p.m. light). Four groups of five mice each were formed and special care was taken to include in the same group animals with similar initial levels of plasma lipids and glucose as well as body weight. No randomization was needed since all mice had identical genetic backgrounds. Three out of four groups received the lavender extract encapsulated in 10% maltodextrin mixed homogeneously with a standard chow diet (#4RF25, Mucedola srI, Milan, Italy) in the following doses: The first group (Lav 1×) received a daily dose of 21.1 mg/kg, the second group (Lav 10×) received a daily dose of 211 mg/kg, and the third group (Lav 100×) received a daily dose of 2110 mg/kg. The fourth group (placebo) received the equivalent amount of the same standard chow diet for the duration of the experiment. KEK and EZ were aware of group allocation during the course of the study. The rest of the investigators performed experiments and analyzed data in a blinded fashion. All animals were individually caged during the course of the study. No animal was excluded from the study. Sample size was determined based on the desired power of statistical analysis, using an online statistical tool (http://www.stat.ubc.ca/~rollin/stats/ssize/n2.html, accessed on 10 December 2024) using mu1 = 0.75, mu2 = 1, σ = 0.1, a = 0.05, and desired power of analysis = 0.95. All animal experiments were conducted according to the EU guidelines of the Protocol for the Protection and Welfare of Animals. The work was authorized by the Laboratory Animal Centre committee of the University of Patras Medical School and the Veterinary Authority of the Prefecture of Western Greece (Authorization number ΠΔΕ/ΔΚ/44966/198 approved on 20 February 2023). The proper implementation of the protocol was monitored by the facility veterinarian as well as ZV and KEK.

### 2.5. Calculation of the Appropriate Dosage

The dose was calculated according to the mean human tolerated dose of other lavender extracts reported in the literature [17], which was estimated at 120 mg daily, using the following equation [35]:

$$Animaldose=Km(human)\times \frac{HED}{Km(animal)}$$

where *HED* stands for Human Estimated Dose expressed in mg/Kg.

Every week during the study, the total amount of the lavender extract used to treat each mouse in the treated group was adjusted, based on body weight changes.

### 2.6. Diet Preparation

The standard chow diet for mice was mixed to homogeneity with the appropriate amount of dried lavender extract, which was produced as described above. The total daily dose of extract was mixed with the total daily amount of food that the mice were consuming. Food was given to the mice daily and its complete consumption ensured that animals received the calculated dose of extract. The placebo group was fed normal chow diet.

### 2.7. Body Weight Measurement

On day 0 (baseline) and day 30 (end of study), the mice were fasted for 16 h and weighed on a Mettler^®^ precision balance.

### 2.8. Intraperitoneal Glucose Tolerance (GTT)

The mice were fasted for 16 h. GTT was performed, and blood glucose was determined at the indicated timepoints. Biochemical determination of plasma glucose levels was performed spectrophotometrically with a DiaSys Glucose FS kit (cat# 125009910021, Diagnostic Systems GmbH, Holzheim, Germany) according to the manufacturer’s instructions.

### 2.9. Determination of Plasma, Lipoprotein, and Liver Lipid Levels

Biochemical determination of plasma total cholesterol and triglyceride levels was performed spectrophotometrically with DiaSys Cholesterol FS kit (cat# 113009910021, Diagnostic Systems GmbH, Holzheim, Germany) and DiaSys Triglycerides FS kit (cat# 157109910021, Diagnostic Systems GmbH, Holzheim, Germany) respectively, according to the manufacturers’ instructions. For hepatic lipid determination, mice were euthanized at the end of the treatment period and liver samples were collected. Hepatic triglyceride levels were measured as described previously [36].

### 2.10. Isolation of Intact Mitochondria

Mitochondria from brown adipose tissue (BAT) and white adipose tissue (WAT) were isolated, as described previously [37]. The protein concentration of each mitochondrial sample was determined using the DCTM protein assay kit (catalog number 500-0116; Bio-Rad, Hercules, CA, USA).

### 2.11. Determination of Liver Enzyme Levels

The determination of liver enzyme levels aspartate aminotransferase (AST), alanine aminotransferase (ALT), and gamma-glutamyl transferase (γ-GT) was performed spectrophotometrically using a Siemens Dimension EXL 200 Integrated Chemistry System (Siemens, Munich, Germany).

### 2.12. Fractionation and Purification of Plasma Lipoproteins by KBr Density Gradient Ultracentrifugation

Following 16 h of fasting, plasma samples were isolated from the groups of mice. Pooled plasma of 0.4 mL from each mouse group was fractionated by KBr density gradient ultracentrifugation, over a 4 mL KBr (Sigma-Aldrich, St. Louis, MO, USA) gradient, as described previously [38].

### 2.13. Western Blot Analysis

For the semiquantitative measurement of murine Apolipoprotein A2 (ApoA2), Apolipoprotein A1 (ApoA1), Apolipoprotein E (ApoE), Apolipoprotein C1 (ApoC1), Apolipoprotein C2 (ApoC2), Apolipoprotein C3 (ApoC3), and Apolipoprotein B (ApoB) in lipoprotein fractions, western blot analysis was performed using the following antibodies: goat anti-ApoA2 (cat# K34001G, Meridian Life Science, Inc., Memphis, Tennessee, USA), rabbit anti-ApoA1 (cat# ES4169, ELK Biotechnology, Denver, CO, USA), rabbit anti-ApoE (E7X2A) (cat# 49285, Cell Signaling, Danvers, MA, USA), goat anti-ApoC1 (cat# K74110G, Meridian Life Science, Inc., Memphis, TN, USA), goat anti-ApoC2 (cat# K59600R, Meridian Life Science, Inc., Memphis, TN, USA), rabbit anti-ApoC3 (cat# ab55984, Abcam, Cambridge, UK and cat# PAB5869, Abnova, Taipei, Taiwan), as primary and rabbit anti-goat IgG-HRP (cat# sc-2768, Santa-Cruz Biotechnology, Inc., Dallas, TX, USA) and goat anti-rabbit IgG-HRP (cat#7074, Cell Signaling, Danvers, MA, USA), as secondary. For the detection of ApoB, a goat anti-ApoB-48/100 HRP (cat# K34005G, Meridian Life Science, Inc., Memphis, TN, USA) was used. Western blotting for cytochrome C (CytC), uncoupling protein 1 (Ucp1), and Cytochrome C oxidase subunit 4 (Cox4) was performed using rabbit anti-mouse antibodies (cat# 4272, Cell Signaling, Danvers, MA, USA; cat# 72298, Cell Signaling, Danvers, MA, USA; and cat# 4844, Cell Signaling, Danvers, MA, USA, respectively). SDS-PAGE (sodium dodecyl sulfate-polyacrylamide gel electrophoresis) of pure mitochondrial extracts was performed using 6 g protein per sample for BAT and 15 g protein per sample for WAT. Semiquantitative determination of the relative protein amounts was performed by Image J free software (Fiji, Version 1.52a, Wayne Rasband, National Institute of Mental Health, Bethesda, MD, USA).

### 2.14. Determination of the Antioxidant Potential of High-Density Lipoprotein (HDL)

For the assessment of the antioxidant capacity of HDL, a modification of the dihydrorhodamine 123 method was used, as described previously [39]. The oxidation rate of dihydrorhodamine in the presence of HDL was assessed. HDL from the placebo group was used as control. The oxidation rate was calculated for each sample as the mean slope for the linear regression of fluorescence intensity between 10 and 60 min of quadruplicates and expressed as fluorescence units per minute: the lower the slope, the lower the substrate oxidation rate.

### 2.15. Effects of HDL on Lipopolysaccharide (LPS)-Induced Inflammation in RAW 264.7 Cells

The effects of the HDL, isolated from the mice, on inflammation were assessed in RAW 264.7 macrophage cell line, as described previously [38].

### 2.16. Statistical Analysis

All data sets were tested using the Kolmogorov–Smirnov and the Shapiro–Wilk tests and were treated with parametric (*p* > 0.1) or non-parametric tests (*p* < 0.1) according to their deviation from normality. No outliers were spotted and all datapoints were included in the analyses. Data were reported as mean ± Standard Error of the Mean (SEM) and statistically significant *p*-value is set at <0.05. * = *p* value < 0.05, ** = *p* value < 0.005 and *** = *p* value < 0.0005. All statistical tests were performed using the GraphPad Prism 8.0.1 software.

## 3. Results

### 3.1. Effect of Lavender Extract on Body Weight

**Figure 1:**
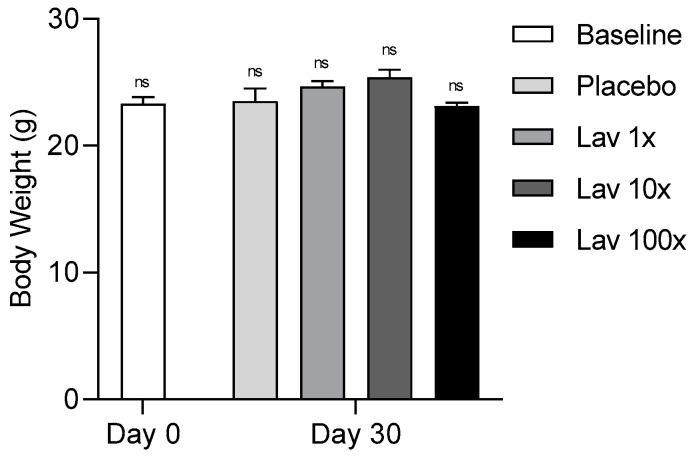
Body weight levels of mice at the beginning and end of the study. Data were analyzed using two-way ANOVA and are presented as Mean ± SEM. Baseline refers to all animals at the beginning of the study (*ns*, non-significant).

To determine the effects of lavender extract treatment on animal body weight, animals were weighed after 16 h of fasting immediately prior to treatment initiation (Baseline) and at the end of the study. As shown in Figure 1, no apparent effect on the body weight of mice was noted following 30 days of treatment with the different doses of our novel lavender extract.

### 3.2. Effect of Lavender Extract on Liver Cholesterol and Triglyceride Levels and Plasma Liver Enzyme Levels

In order to evaluate the safety of lavender extract administration, we tested liver toxicity in the treated mice by measuring hepatic lipid content and the levels of three main liver enzymes, namely alanine aminotransferase—ALT (SGPT), aspartate aminotransferase—AST (SGOT), and gamma-glutamyl transferase (γ-GT), in the plasma of the experimental animals.

**Figure 2:**
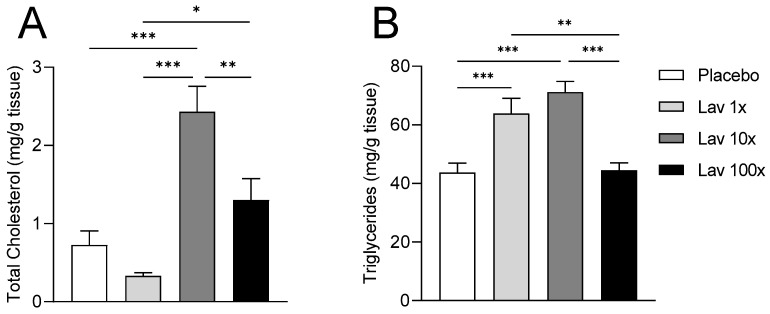
Determination of hepatic lipid levels. Panel (**A**) indicates the hepatic cholesterol content and panel (**B**) the hepatic triglyceride content per gram of liver tissue isolated from all groups of mice. Data were analyzed using one-way ANOVA and are presented as Mean ± SEM. * = *p* value < 0.05, ** = *p* value < 0.005 and *** = *p* value < 0.0005.

The 1× dose triggered only a mild increase in AST and γ-GT liver enzymes while it lowered ALT, even though it increased hepatic triglyceride content compared to the placebo group (Figure 2A,B, and Table 1). Similarly, hepatic lipid content determination showed that a 10× administration of lavender extract resulted in significantly elevated cholesterol and triglyceride levels (Figure 2A,B) compared to the other groups, but did not negatively impact the levels of the liver enzymes (Table 1). Finally, the 100× dose did not alter either cholesterol or triglyceride levels in the liver, but resulted in a moderate elevation in liver enzymes (Figure 2A,B and Table 1).

### 3.3. Effect of Lavender Extract on Plasma and Lipoprotein Lipid Levels

Plasma lipid levels were determined at the beginning (Baseline) and at the end of the study after 16 h of fasting. Lipoprotein lipid levels were determined only at the end of the study after the mice were sacrificed.

**Figure 3:**
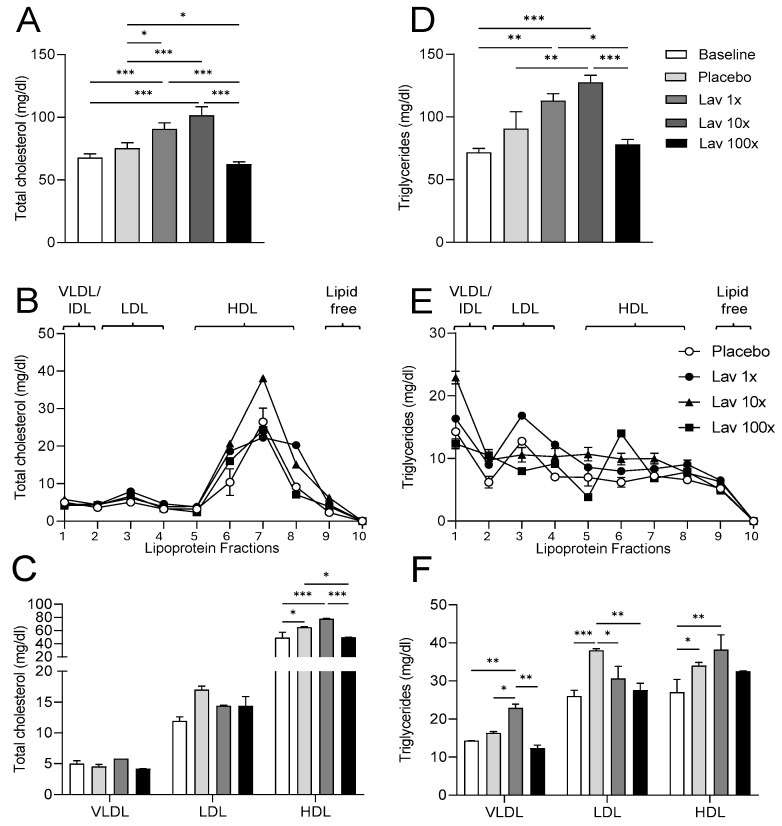
Plasma total cholesterol (**A**) and triglyceride (**D**) levels, lipoprotein cholesterol (**B**,**C**) and triglyceride (**E**,**F**). Data were analyzed using one-way ANOVA (**A**,**D**) and two-way ANOVA (**C**,**F**) and are presented as Mean ± SEM. VLDL, very low density lipoprotein; IDL, intermediate density lipoprotein; LDL, low-density lipoprotein; HDL, high density lipoprotein. * = *p* value < 0.05, ** = *p* value < 0.005 and *** = *p* value < 0.0005.

Our data indicate that the administration of doses 1× and 10× resulted in a significant increase of plasma cholesterol. Additionally, the 10× dose led to a significant increase in triglyceride levels, while the 100× dose did not affect plasma triglycerides, while it significantly decreased plasma cholesterol as compared to the placebo group (Figure 3A,D). Further analysis of lipoprotein lipid levels showed that the cholesterol increase observed with 1× and 10× doses was mainly due to HDL cholesterol levels, while the 100× dose did not increase HDL-C levels compared to the placebo (Figure 3B,C). The 1× dose led to a significant increase in LDL and HDL triglyceride content, while the 10× dose affected VLDL and HDL triglyceride levels (Figure 3E,F).

### 3.4. Effect of Lavender Extract on the Apolipoprotein Content of Plasma Lipoproteins

**Figure 4:**
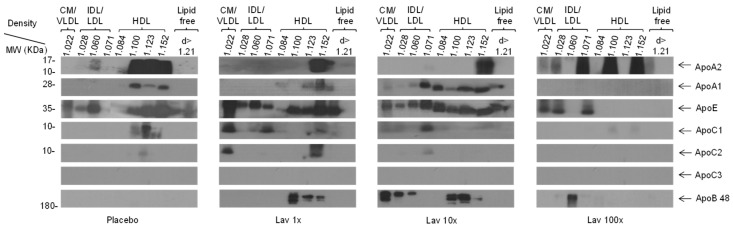
Representative Western blot analysis of apolipoprotein content of plasma lipoproteins fractions isolated by UCF. ApoA2, Apolipoprotein A2; ApoA1, Apolipoprotein A1; ApoE, Apolipoprotein E; ApoC1, Apolipoprotein C1; ApoC2, Apolipoprotein C2; ApoC3, Apolipoprotein C3; ApoB-48, Apolipoprotein B-48; CM, Chylomicron; VLDL, very low-density lipoprotein; IDL, intermediate density lipoprotein; LDL, low-density lipoprotein; HDL, high-density lipoprotein; UCF, ultracentrifugation.

Analysis of the apolipoprotein content of plasma lipoproteins showed that in the placebo group significant amounts of ApoA1, ApoA2, ApoC1, and ApoE were primarily present in the HDL fractions, as expected (Figure 4). ApoΕ in particular was present in the VLDL/IDL/LDL fractions. In the 1× and 10× dose groups, treatment with the lavender extract decreased the levels of apolipoprotein A2 while it increased the levels of apolipoproteins E, B and C1 compared to the placebo group (Figure 4). In addition, administration of the 1× dose led to an increase in the levels of apolipoprotein C2, while it did not affect the levels of apolipoprotein A1 compared to the placebo. In contrast, treatment with the 10× dose did not affect the levels of apolipoprotein C2, while it appeared to increase the levels of apolipoproteins A1 and E relative to the placebo. Finally, administration of the 100× dose led to a notable redistribution of apolipoprotein A2, which now also appears in small LDL fractions as well as VLDL (Figure 4). This dose also resulted in the disappearance of apolipoprotein B from the HDL fractions and its redistribution in considerably lower amounts in the LDL, compared to the 1× and 10× treated groups. In addition, the 100× dose led to a reduction in all other apolipoproteins relative to both the placebo and the other two doses.

### 3.5. Effect of Lavender Extract on the Anti-Inflammatory and Antioxidant Capacity of HDL

**Figure 5:**
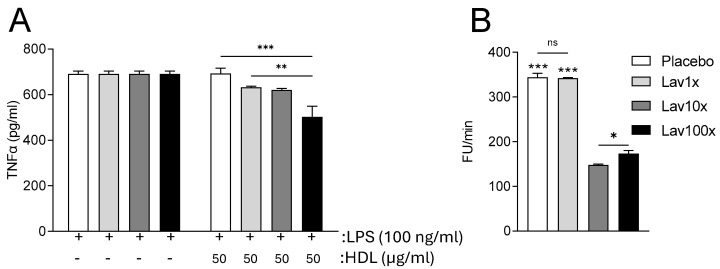
Anti−inflammatory (**A**) and antioxidant (**B**) capacity of HDL isolated from the plasma of all groups of mice. Panel (**A**) indicates the effects of HDL on LPS (100 ng/mL)−induced TNFα release from cultured RAW 264.7 macrophages. Panel (**B**) indicates DHR oxidation rate after the addition of 5 µg HDL−cholesterol over the course of 60 min. Data were analyzed using two−way ANOVA (**A**) and one−way ANOVA (**B**) and are presented as Mean ± SEM. * = *p* value < 0.05, ** = *p* value < 0.005 and *** = *p* value < 0.0005.

Based on our previous observations [40] that the apolipoprotein and lipid content of HDL particles modulated their functionality, next we proceeded to determine the anti-inflammatory and antioxidant capacity of the plasma HDL isolated from experimental animals. Our results show that the HDL, isolated from mice treated with the 100× dose of the lavender extract, possessed the best anti-inflammatory capacity compared to the other groups and the placebo group, leading to significantly reduced TNFα secretion, following LPS challenge, in cultured RAW 264.7 macrophages (Figure 5A).

When we tested HDL from the different mouse groups for its antioxidant capacity, we observed a significant reduction of DHR oxidation following the addition of HDL isolated from both 10× and 100× groups. Specifically, the administration of 10× and 100× doses of lavender extract led to the production of HDL particles with significantly increased antioxidant capacity, with the 10× dose showing the highest capacity compared to all other doses and the placebo (Figure 5B).

### 3.6. Effect of Lavender Extract on Fasting Glucose Levels and Glucose Tolerance

To evaluate the effects of lavender extract on the glycemic profile of the experimental animals, we performed a classical glucose tolerance test (GTT) along with a measurement of fasting plasma glucose levels.

**Figure 6:**
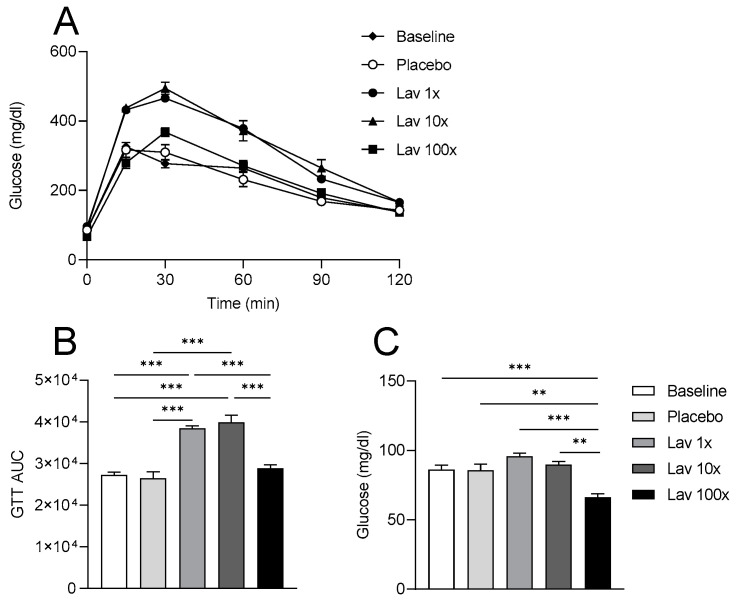
Effect of lavender extract on the glycemic profile of the experimental animals. (**A**) Glucose tolerance test (GTT) at the beginning (Baseline) and at the end of the study. (**B**) Mean area under the curve (AUC) for each group at the beginning (Baseline) and at the end of the study calculated for GTT curves. Panel (**C**) indicates the fasting plasma glucose levels at the beginning (Baseline) and at the end of the study. Data were analyzed using one-way ANOVA (**A**,**C**) and are presented as Mean ± SEM. ** = *p* value < 0.005 and *** = *p* value < 0.0005.

From the glucose tolerance test, performed at the beginning (Baseline) and at the end of the study, we noted a significant decrease in glucose tolerance with the administration of 1× and 10× doses of lavender extract. Nevertheless, the administration of the 100× dose had no apparent effect on glucose tolerance, compared to the placebo (Figure 6A,B). Fasting plasma glucose levels also indicated that the administration of a 100× dose of lavender extract led to reduced glucose levels, compared to all other groups (Figure 6C).

### 3.7. Effect of Lavender Extract on Mitochondrial Metabolic Activation of Brown and White Adipose Tissue

Next, we proceeded to the evaluation of adipose tissue mitochondrial metabolic activation. For this purpose, intact mitochondria were isolated from brown (BAT) and white (WAT) adipose tissue harvested from the experimental animals. Mitochondrial fractions were then analyzed by Western blot for their uncoupling protein 1 (Ucp1), cytochrome C (CytC), and cytochrome C oxidase subunit 4 (Cox4) levels.

**Figure 7:**
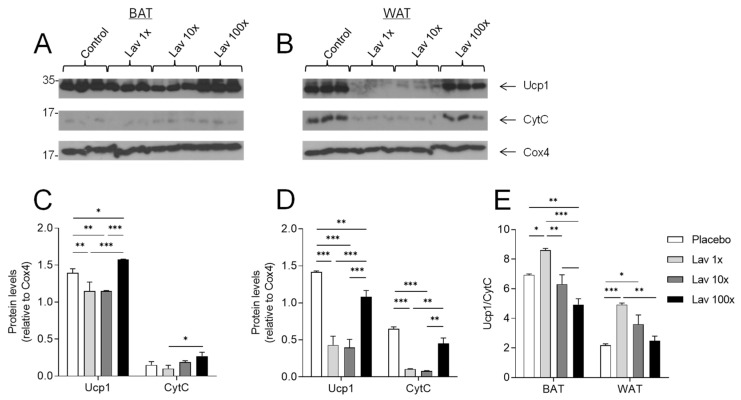
Representative Western blotting analysis and semiquantitative determination of Ucp1 and CytC relative to Cox4 in mitochondrial extracts (Panels (**A**,**B**)). Panels (**C**,**D**) indicate mitochondrial Ucp1 and CytC levels corrected for Cox4 levels, in BAT and WAT respectively. Panel E indicates the relative Ucp1/CytC ratio in BAT and WAT respectively. Data were produced from the same blots probed with the indicated antibodies. Statistical analysis was performed using a two-way ANOVA and are presented as mean ± SEM. Semiquantitative determination of the relative protein amounts was performed by ImageJ free software (Fiji, Version 1.52a, Wayne Rasband). WAT, white adipose tissue; BAT, brown adipose tissue; Ucp1, uncoupling protein 1; CytC, cytochrome C; Cox4, cytochrome C oxidase subunit 4. * = *p* value < 0.05, ** = *p* value < 0.005 and *** = *p* value < 0.0005.

Analysis of isolated BAT mitochondria from mice treated with 1× and 10× doses suggested decreased metabolic activation compared to the placebo and the 100× dose groups, mainly due to decreased non-shivering thermogenesis, as indicated by Ucp1 levels (Figure 7A,C). In contrast, administration of the 100× dose resulted in increased BAT mitochondria oxidative phosphorylation and non-shivering thermogenesis, as indicated by the elevated Ucp1 and CytC in relation to Cox4 levels (Figure 7A,C, E).

A similar analysis of WAT mitochondrial fractions showed that all three doses (1×, 10× and 100×) of lavender extract significantly decrease WAT mitochondrial metabolic activation, compared to the placebo, as indicated by the decreased levels of both Ucp1 and CytC levels. Nevertheless, the 100× dose administration of the lavender extract led to significantly increased levels of both Ucp1 and CytC, compared to the other two doses, indicating enhanced WAT mitochondrial oxidative phosphorylation and non-shivering thermogenesis, compared to the two groups treated with 1× and 10× doses (Figure 7B,D,E).

## 4. Discussion

The benefits of lavender for human health as well as its use for medicinal purposes have been known for centuries [2]. In addition to its antifungal, antibacterial [3,4,5], anticholinergic, relaxing [2] and antidepressant [7] properties, which have been extensively studied, lavender has an important effect on both the cardiovascular system and the management of blood glucose levels. Preclinical and clinical studies indicated that the use of lavender essential oil can lower blood pressure [10,14] and improve coronary circulation by reducing aortic cholesterol content and atherosclerotic plaque formation, thus providing an antiatherogenic effect [11,12] Furthermore, the well-known antioxidant properties of lavender oil and solid waste were confirmed by the high phenolic content and antioxidant capacity of the extract and dried extract determined in this research work.

As part of our ongoing efforts to valorize aromatic herbs in the pursuit of circular economy and environmental benefits, in the present study we tested the efficacy and potential toxicity of a maltodextrin encapsulated lavender extract isolated from the leaves of the plant after essential oil was removed. Among other benefits, encapsulation was essential to minimize lavender odor.

Our results summarized in Table 2 show that none of the doses of lavender extract used affect the body weight of the experimental animals. From the measurement of blood lipids, it appears that the 1× and 10× doses trigger a mild increase in plasma cholesterol. This increase does not appear to result in adverse effects for health and rather it may be beneficial since fractionation of plasma lipoproteins and measurement of their lipid content showed that this increase in plasma cholesterol was mainly due to the increase in HDL cholesterol. In contrast, the 100× dose appears to reduce plasma cholesterol levels due to a reduction in the cholesterol content of all lipoprotein fractions. When plasma triglyceride levels were measured, it was found that the 1× and 10× doses increase triglyceride levels with the most significant increase in VLDL fraction at the 10× dose and LDL fraction at the 1× dose. Furthermore, it appears that the 100× dose does not affect plasma triglyceride levels but causes a redistribution of triglycerides with a decrease in VLDL triglycerides and an increase in HDL triglycerides.

Apolipoprotein analysis of plasma lipoproteins showed that administration of lavender extract at any of the three doses increased apolipoprotein B levels. This increase was much more pronounced at the 1× and 10× doses where APOB was also present in the HDL fractions, while at the 100× dose it was much milder and APOB was only present in LDL. Recent studies have shown that apolipoprotein B levels follow a direct and linear correlation with increased cardiovascular risk [25]. At the 1× and 10× doses the increase in APOB levels is consistent with the corresponding increase in serum triglycerides, suggesting a possible significant increase in residual cardiovascular risk at these doses due to increased number of triglyceride-rich lipoproteins. Moreover, at these two doses, APOB appears in density fractions where HDL particles are expected, suggesting the possible presence of very small-dense LDL particles, which are particularly atherogenic.

However, the 10× dose appears to increase levels of the atheroprotective apolipoprotein A1, an effect that mitigates the increased residual risk associated with increased presence of APOB-containing triglyceride-rich lipoproteins. ApoA1 promotes the de novo biogenesis of HDL and this may explain the increased HDL-C levels obtained in the 1× and 10× groups. On the other hand, the 10× dose slightly increases the levels of ApoC1, an apolipoprotein that is associated with an adverse effect on lipoprotein profiles including inhibition of lipoprotein lipase (LpL) and lecithin–cholesterol acyltransferase (LCAT) activities, with respective negative effects on lipid profiles [26]. Contrasting these findings with the two lower doses, the 100× dose triggers only a mild increase in plasma APOB levels exclusively in the LDL fractions and is not accompanied by any increase in cholesterol or triglyceride levels of these fractions, indicating the lack of a possible proatherogenic effect of the 100× dose.

The significant change in lipid and apolipoprotein content observed in HDL lipoprotein fractions combined with evidence in the literature for potential antioxidant and anti-inflammatory [3,4,5] properties of lavender led us to measure the anti-inflammatory and antioxidant activity of isolated HDL from the plasma from experimental animals treated with the various doses of our lavender extract. Measurement of HDL anti-inflammatory activity showed that the 100× dose of lavender extract led to improved HDL anti-inflammatory activity compared to the placebo treated group, as indicated by the significant reduction in the release of the proinflammatory cytokine TNFα. Furthermore, following measurement of HDL antioxidant activity, it appears that the 10× and 100× doses of our lavender extract led to better antioxidant capacity. Overall, administration of our lavender extract in our mouse model is associated with notably improved HDL functionality.

The positive effects of lavender extract treatment on HDL functionality led us to hypothesize that our extract may have a significant benefit on plasma glucose homeostasis [41]. Data in the literature on the effect of lavender essential oil on glucose homeostasis are conflicting, with some studies supporting beneficial effects in rodents [15,17] and others showing no effect on the glycemic profile of humans [18]. Our own analysis showed that the administration of our lavender extract at the doses of 1× and 10× does not lead to a change in fasting glucose levels, but rather is associated with reduced post-prandial plasma glucose clearance consistent with reduced glucose tolerance. However, in agreement with the notable increase in HDL antioxidant and anti-inflammatory activity at the 100× dose, treatment with this dose lowers fasting glucose levels and maintains normal blood glucose tolerance.

Since glucose tolerance is often associated with obesity, and obesity at the molecular level is linked to reduced levels of mitochondrial metabolic activity, in the next set of experiments we performed a biochemical characterization of mitochondrial metabolic activation of purified mitochondria from WAT and BAT of the mice treated with the three different doses of our lavender extract. This analysis showed that the administration of the lavender extract in the 1× and 10× groups resulted in a significant decrease in non-shivering thermogenesis in BAT. However, treatment with the 100× dose led to a significant increase in thermogenesis in this tissue, as shown by the levels of Ucp1 compared to the placebo group, while not affecting ATP production through oxidative phosphorylation in this tissue. In contrast, administration of all three doses of lavender extract led to a significant reduction in WAT mitochondrial non-shivering thermogenesis as evidenced by the reduction in mitochondrial Ucp1 levels, with the greatest reduction observed at the 1× and 10× doses. On the other hand, ATP production via oxidative phosphorylation was reduced only at 1× and 10× doses compared to the placebo. Overall, it appears that out of the three doses tested, administration of 100× lavender extract appears to have superior effects compared to the other two, as it promotes thermogenesis at the expense of ATP production in both BAT and WAT.

To determine any potential adverse effects of our extract on liver function, we measured hepatic cholesterol and triglyceride content, as well as ALT, AST, and γ-GT levels in the plasma of experimental animals. γ-GT is a molecular marker of hepatic oxidative stress which is a crucial process for the development of NASH. Any value ≤4 is considered physiological. On the other hand, ALT and AST are more sensitive markers of acute liver toxicity. Treatment with the 10× dose increased both liver cholesterol and triglyceride levels, while the 1× dose increased only liver triglyceride levels. However, ALT and γ-GT levels at these doses remained normal, while only AST showed a moderate increase. In contrast, at the 100× dose, although no significant change was identified in hepatic cholesterol and triglyceride levels, a doubling of ALT (SGPT) and AST (SGOT) levels and a very slight increase in γ-GT were observed compared to the placebo group. Nevertheless, this increase in hepatic enzyme levels is considered mild for the safety of extract even at the 100× dose. Based on the very long experience we have with statins, which can also raise hepatic enzymes, changes up to three-fold are considered acceptable and do not pose a threat for health or a reason for statin discontinuation. However, with respect to our extract, longer treatment periods may be needed to clarify this point and show whether this increase is transient, as it often happens with popular medications like statins [42].

## 5. Study Limitations

A limitation of our study is that all animals tested were males and approximately 24 weeks old. We have no data on female mice or mice of different ages to evaluate the impact of sex or age on the outcomes of our study. Another limitation is that we did not investigate the effects of the extract under different diets (for example high-fat diet) since in the present study our goal was to investigate the effects of our lavender extract in a normolipidemic condition and under a standard diet representing a person with normal dietary habits. Finally, additional toxicity tests, including organ toxicity, are needed for a complete characterization of the safety profile of the extract.

## 6. Conclusions

In conclusion, administration of our *Levander stoechas* extract results in pleiotropic beneficial effects on lipid and lipoprotein profiles, as well as glucose and adipose tissue metabolism, with these effects appearing to be dose-dependent. Based on our results, the 100× dose, which, in mice corresponds to 2110 mg per kg body weight, has the most beneficial effects (Table 2). Furthermore, no serious safety concerns are raised for this dose aside from a modest increase in liver enzyme levels detected in the blood.

## Figures and Tables

**Table 1:** Plasma liver enzyme levels.

Liver Enzyme (IU/L)	Placebo	Lav 1×	Lav 10×	Lav 100×
Alanine Aminotransferase—ALT (SGPT ^1^)	60.5	40.0	41.0	118.0
Aspartate Aminotransferase—AST (SGOT ^2^)	178.0	230.0	226.0	345.0
Gamma-Glutamyl Transferase—γ-GT	3.5	4.0	2.0	4.0

^1^ SGPT, Serum Glutamic Pyruvic Transaminase; ^2^ SGOT, Serum Glutamic Oxaloacetic Transaminase.

**Table 2:** Comparison of parameters tested between groups, in relation to the placebo.

Study Groups/Parameters	1×	10×	100×
Body Weight	=	=	=
Plasma cholesterol	+	+++	-
Plasma triglycerides	=	++	=
Cholesterol in VLDL	=	=	=
Cholesterol in LDL	=	=	=
Cholesterol in HDL	+	+++	=
Triglycerides in VLDL	=	++	=
Triglycerides in LDL	+++	=	=
Triglycerides in HDL	+	++	=
Anti-inflammatory effect of HDL	=	=	+++
Antioxidant activity of HDL	=	+++	+++
Liver cholesterol	=	+++	=
Hepatic triglycerides	+++	+++	=
Transaminase ALT (SGPT)	-	-	+
Transaminase AST (SGOT)	+	+	+
γ-Glutamyltransferase (γ-GT)	=	-	=
Basal state fasting plasma glucose	=	=	- -
Glucose tolerance	- - -	- - -	=
Ucp1 (BAT)	- -	- -	+
Mitochondrial Cytc (BAT)	=	=	=
Ucp1/Cytc (BAT)	+	=	- -
Ucp1 (WAT)	- - -	- - -	- -
Mitochondrial Cytc (WAT)	- - -	- - -	=
Ucp1/Cytc (WAT)	+++	+	=
ApoA2	--	--	= #
ApoA1	=	+	-
ApoE	=	=	-
ApoC1	+	+	-
ApoC2	+	=	-
ApoC3	=	=	=
ApoB48	++	++	+

=: no significant change, -: significant decrease corresponding to *p* value < 0.05, - -: significant decrease corresponding to *p* value < 0.005, - - -: significant decrease corresponding to *p* value < 0.0005, +: significant increase corresponding to *p* value < 0.05, ++: significant increase corresponding to *p* value < 0.005, +++: significant increase corresponding to *p* value < 0.0005, #: signifies redistribution in the case of lipoproteins.

## Data Availability

The raw data supporting the conclusions of this article were submitted to the Journal during the review process. Further inquiries can be directed to the corresponding author.

## Supplementary Materials

The following supporting information can be downloaded at: https://www.mdpi.com/article/10.3390/nu17010076/s1, Table S1: Physicochemical properties of dried extract; Table S2: Antioxidant properties of extract and dried extract.
