# Supplementary material for: Effect of a Novel Lavender Extract on Plasma Lipid and Lipoprotein Metabolism, Glucose Tolerance and Adipose Tissue Metabolic Activation: A Preclinical Safety and Efficacy Study
Source: Nutrients. 2024 Dec 28;17(1):76. doi: 10.3390/nu17010076 (PMC11722746; doi:10.3390/nu17010076)
Supplement: Supplementary file 1 [file nutrients-17-00076-s001.zip › nutrients-3377367-supplementary.pdf]

**Table S1.** Physicochemical properties of dried extract (avg±SD, n=3)

| | |
|---|---|
| Moisture Content (% Wet Basis) | 4.6 ± 0.3 |
| Bulk Density (mg/mL) | 363±1.5 |
| Hygroscopicity @ 25°C, 75% RH (g Water/100 g Powder) | 9.7 ± 0.9 |
| Water Activity ($a_w$) | 0.33 ± 0.02 |

**Table S2.** Antioxidant properties of extract and dried extract (avg±SD, n=3)

| **Properties** | **UAE extract** | **Spray-dried UAE extract with 10% maltodextrin** |
|---|---|---|
| Total Phenolic Content (mg GAE/g Dry Lavender) | 21 ± 4 | 17 ± 2 |
| % Antioxidant Capacity | 89 ± 1 | 84 ± 3 |
| FRAP (μmol TPTZ/L) | 12 ± 1 | 9 ± 1 |
